# Platelet Activation and Anti-Phospholipid Antibodies Collaborate in the Activation of the Complement System on Platelets in Systemic Lupus Erythematosus

**DOI:** 10.1371/journal.pone.0099386

**Published:** 2014-06-12

**Authors:** Christian Lood, Helena Tydén, Birgitta Gullstrand, Gunnar Sturfelt, Andreas Jönsen, Lennart Truedsson, Anders A. Bengtsson

**Affiliations:** 1 Department of Clinical Sciences Lund, Section of Rheumatology, Lund University and Skåne University Hospital, Lund, Sweden; 2 Department of Laboratory Medicine Lund, Section of Microbiology, Immunology and Glycobiology, Lund University, Lund, Sweden; Centro Cardiologico Monzino IRCCS, Italy

## Abstract

Anti-phospholipid (aPL) antibodies are important contributors to development of thrombosis in patients with the autoimmune rheumatic disease systemic lupus erythematosus (SLE). The underlying mechanism of aPL antibody-mediated thrombosis is not fully understood but existing data suggest that platelets and the complement system are key components. Complement activation on platelets is seen in SLE patients, especially in patients with aPL antibodies, and has been related to venous thrombosis and stroke. The aim of this study was to investigate if aPL antibodies could support classical pathway activation on platelets *in vitro* as well as in SLE patients. Furthermore, we investigated if complement deposition on platelets was associated with vascular events, either arterial or venous, when the data had been adjusted for traditional cardiovascular risk factors. Finally, we analyzed if platelet complement deposition, both C1q and C4d, was specific for SLE. We found that aPL antibodies supported C4d deposition on platelets *in vitro* as well as in SLE patients (p = 0.001 and p<0.05, respectively). Complement deposition on platelets was increased in SLE patients when compared with healthy individuals (p<0.0001). However, high levels of C4d deposition and a pronounced C1q deposition were also seen in patients with rheumatoid arthritis and systemic sclerosis. In SLE, C4d deposition on platelets was associated with platelet activation, complement consumption, disease activity and venous (OR = 5.3, p = 0.02), but not arterial, thrombosis, observations which were independent of traditional cardiovascular risk factors. In conclusion, several mechanisms operate in SLE to amplify platelet complement deposition, of which aPL antibodies and platelet activation were identified as important contributors in this investigation. Complement deposition on platelets was identified as a marker of venous, but not arterial thrombosis, in SLE patients independently of traditional risk factors and aPL antibodies. Further studies are needed to elucidate the role of complement deposition on platelets in development of venous thrombosis.

## Introduction

Systemic lupus erythematosus (SLE) is an autoimmune rheumatic disease characterized by systemic inflammation affecting several organ systems including joints, kidney, skin and central nervous system [1]. SLE patients have a highly increased cardiovascular morbidity and mortality which can only be partly explained by traditional risk factors [2], [3], [4], [5]. Anti-phospholipid (aPL) antibodies are a group of phospholipid-binding autoantibodies with overlapping, but partly different specificities. There are three main aPL tests used in clinical practice; anti-cardiolipin (aCL) antibodies, anti-beta 2 glycoprotein I (aB2GPI) antibodies and lupus anticoagulans (LA). Positivity in one or more of those assays is associated with development of venous thrombosis and stroke [6], [7], [8], [9]. The underlying mechanism of aPL antibody-mediated thrombosis is not fully understood. It is known that aPL antibodies are able to bind to platelets and amplify platelet activation and aggregation through the p38 MAPK signaling pathway [10], [11], [12], [13], [14], [15]. Furthermore, investigations in complement deficiency, both in mice and human, suggest that classical pathway activation of the complement system is essential in development of aPL antibody-mediated thrombosis [16], [17], [18], [19], [20], [21]. Thus, even though the exact underlying mechanism for aPL antibody-mediated development of thrombosis is still not known, existing data suggest that two of the components behind the pro-thrombotic effects are platelets and the complement system.

Data from our group and from others have previously demonstrated that SLE patients have increased complement activation on platelets, especially patients with aPL antibodies [22], [23], [24]. It is known that some aPL antibodies have complement-fixing activity and allow complement activation through the classical pathway [25]. However, whether aPL antibodies support complement activation specifically on platelets is not known. In addition, complement activation on platelets may be caused by platelet activation and subsequent exposure of C1q binding epitopes on the activated platelet cell surface [23], [26]. Currently, it is unclear which of these mechanisms, autoantibody-mediated complement activation or direct binding of C1q due to platelet activation, is operating in SLE to increase platelet complement deposition.

Complement deposition on platelets has been seen in cases of individuals with stroke, but is otherwise thought to be specific for SLE [22], [27], although studies have not been extensive in other chronic inflammatory diseases. In SLE, increased C4d deposition on platelets is associated with vascular events [23], [24], [28]. However, there are discrepancies in the literature as to whether it is venous or arterial vascular events which are associated with complement deposition on platelets. In addition it is also important to note that none of the previous investigations adjusted data for traditional cardiovascular risk factors.

The aim of this study was to investigate if aPL antibodies could support classical pathway activation on platelets *in vitro* as well as in SLE patients. Furthermore, in data which had been adjusted to account for traditional cardiovascular risk factors and aPL antibodies, we investigated with which kind of vascular events, arterial or venous, complement deposition on platelets was associated. Finally, we analyzed if deposition of complement factors C1q and C4d on platelets was specific for SLE or also found in disease controls and healthy individuals. In brief we found that aPL antibodies supported activation of the classical pathway of the complement system on platelets by two separate mechanisms; amplification of platelet activation, and by providing complement-fixing antibodies on the platelet surface. Platelet activation was analyzed by flow cytometry measuring platelet P-selectin and CD69 expression. CD69 is constitutively expressed on platelets, but is increased upon activation and is important for platelet aggregation [29], [30]. In SLE patients, deposition on platelets of both complement factor C1q and C4d, was associated with venous, but not arterial, thrombosis when the data was adjusted to account for traditional cardiovascular risk factors and aPL antibodies. These results suggest a possible link between aPL antibodies and development of venous thrombosis through mechanisms involving complement activation on platelets. Finally, complement deposition on platelets was not specific for SLE but high levels of both C1q and C4d on platelets were also found in other disease groups, in particular in patients with rheumatoid arthritis.

## Materials and Methods

### Patients

Patients with SLE (n = 148), rheumatoid arthritis (n = 20) and systemic sclerosis (n = 20) were recruited to participate in studies related to cardiovascular disease at the Department of Rheumatology, Skåne University Hospital, Lund, Sweden. Healthy volunteers (n = 79) were age- and sex-matched as a group to the SLE patients. Patients who had a recent myocardial infarction (within the previous year) but with no diagnosis of chronic inflammatory diseases (n = 39) were recruited at the Department of Cardiology, Skåne University Hospital, Lund, Sweden. The myocardial infarction patients were treated with Warfarin (n = 3), acetylsalicylic acid (n = 34) and the P2Y_12_ receptor inhibitor Clopidogrel (n = 9). Furthermore, all patients with myocardial infarction used different anti-hypertension treatments, including beta blockers (n = 28). An overview of the clinical characteristics of the patients is presented in Tables 1, 2, and 3. Disease activity was assessed using SLEDAI-2K [31]. All but two individuals fulfilled at least four American College of Rheumatology (ACR) 1982 criteria for SLE [32]. These two patients fulfilled three ACR criteria, had a clinical SLE diagnosis with at least two organ manifestations characteristic of SLE, autoimmune phenomena, and no other diagnosis that could better explain the symptoms. The following treatments were used in the SLE cohort at the time of blood sampling: glucocorticoids (n = 98, median dose = 5 mg, range 1–30 mg), hydroxychloroquine (n = 105), azathioprine (n = 32), mycophenolatmofetil (n = 20), methotrexate (n = 13), intravenous immunoglobulins (n = 2), non-steroidal anti-inflammatory drugs (n = 12), acetylsalicylic acid (n = 44) and Warfarin (n = 23). Previous episodes of myocardial infarction, claudicatio intermittens, cerebrovascular incidents (CVI), angina pectoris, deep venous thrombosis or pulmonary embolisms were defined by the Systemic Lupus International Collaborative Clinics/ACR Damage Index (SLICC/ACR-DI) [33]. Traditional cardiovascular risk factors; age, gender, smoking, diabetes, hypertension (systolic blood pressure equal or higher than 140 at the time of blood sampling or hypertensive treatment due to high blood pressure), body mass index (BMI) and LDL levels, were assessed at the visit to the clinic (Table 3). Complement proteins and autoantibodies were measured by routine standard analyses at the Department of Clinical Immunology and Transfusion Medicine, LabMedicin Skåne, Lund, Sweden.

**Table 1:** Distribution of American College of Rheumatology (ACR) 1982 classification criteria for the 148 SLE patients.

ACR criteria, median (range)	5 (3–10)
Malar rash %	52
Discoid rash %	20
Photosensitivity %	56
Oral ulcers %	24
Arthritis %	78
Serositis %	39
Renal disease %	33
Neurological disorder %	6
Hematological manifestations %	55
Leukopenia %	37
Lymphopenia %	24
Thrombocytopenia %	14
Immunology %	69
Anti-dsDNA antibodies %	59
ANA %	98

**Table 2:** Clinical characteristics of the SLE patients.

Disease duration, median (range), years	11 (0–46)
SLEDAI score, median (range)	1.5 (0–18)
Serum C3 (g/L), median (range)	1.02 (0.36–2.46)
Serum C4 (g/L), median (range)	0.15 (0.03–0.58)
Serum C1q (%), median (range)	102 (8–200)
Serum C3dg (mg/L), median (range)	0 (0–25.10)
aCL at visit %	5
aCL titer (GPLU/mL)^a^, median (range)	60 (42–158)
aCL ever %	28
aB2GP1 at visit %	7
aB2GP1 titer (U/mL)^a^, median (range)	28 (16–100)
aB2GP1 ever %	11
Lupus anticoagulans visit %	11
Lupus anticoagulans ever %	11
SLICC/ACR-DI, median (range)	0 (0–8)

a Calculation only done for patients with detectable levels of autoantibodies. Abbreviations: SLEDAI; SLE disease activity index, aCL; anti-cardiolipin antibody, aB2GP1; anti-beta 2 glycoprotein 1 antibody.

**Table 3:** Distribution of traditional cardiovascular risk factors in the different cohorts included in the study.

Patient group	Healthy volunteers	SLE	RA	SSc	MI
Number	79	148	20	20	39
Female %	85	87	75	80	15
Age (median, range)	47 (18–81)	48 (20–82)	56 (28–67)	67 (19–82)	69 (61–77)
LDL concentration (mM), mean and SD	3.16±0.87	3.06±0.95	3.28±0.87	2.91±1.10	2.41±1.08
Smoking %	9	21	30	20	0
Diabetes %	1	3	5	5	15
Hypertension^a^ %	18	43	30	35	69
Body mass index	23.5±3.1	25.5±4.9	26.5±4.2	22.5±3.0	25.7±2.9

a Hypertension was defined as systolic blood pressure equal or higher than 140 at time point of blood sampling or hypertensive treatment due to high blood pressure. Abbreviations: LDL; low-density lipoproteins.

### Ethics statement

The study was approved by the regional ethics board (LU-06014520) and an informed written consent was obtained from all participants.

### Complement deposition on platelets in SLE patients

Blood, collected in sodium-citrate tubes (BD Biosciences Pharmingen, Franklin Lakes, NJ, USA), was centrifuged at 280×g for 10 minutes to obtain platelet-rich plasma (PRP). Platelet purity was routinely analyzed by CD42a expression and was found to be more than 98% (mean: 98.8% (range 98.1–99.4%). Ethylenediaminetetraacetic acid (EDTA) was added to PRP to a final concentration of 10 mM to avoid complement activation during the isolation process, and then the platelets were centrifuged at 1125×g for 10 minutes. The platelets were resuspended in 10 mM N-2-hydroxyethylpiperazine-N′-2-ethanesulfonic acid (HEPES) buffer pH 7.4, containing 145 mM NaCl and 5 mM KCl (HEPES buffer) and incubated with fluorescein isocyanate (FITC)-conjugated anti-C1q antibodies (Dako, Glostrup, Denmark) or antibodies against C4d (Quidel, San Diego, CA, USA) for 30 minutes at room temperature. For detection of C4d, the platelets were washed once in HEPES buffer and then incubated with FITC-conjugated rabbit anti-mouse IgG antibodies (Dako) for an additional 30 minutes at 4°C. The platelets were analyzed by flow cytometry on an Accuri C6 (BD).

### In vitro complement deposition on activated platelets

PRP, 5 µl, was incubated with 5 µM ADP (Chrono-log, Havertown, PA, USA), for 30 minutes at room temperature in phosphate buffered saline pH 7.4 (PBS). The activation was terminated by incubation with 2% paraformaldehyde for 10 minutes. Activated and fixed platelets were isolated by centrifugation at 1125×g for 10 minutes. Purified platelets were resuspended in veronal buffered saline with 0.15 mM Ca^2+^ and 0.5 mM Mg^2+^ (VBS CaMg) containing 10% normal human serum and human IgG (Immuno AG, Vienna, Austria) or anti-cardiolipin IgG antibodies, at a final concentration of 20 µg/ml, (Antibodies-online.com, Atlanta, GA, USA) and incubated for 60 minutes at 37°C to allow complement activation. The platelets were washed once in PBS and incubated with an anti-C4d antibody (Quidel) for 30 minutes followed by a FITC-conjugated rabbit anti mouse IgG antibody (Dako) for an additional 30 minutes at 4°C. The platelets were analyzed by flow cytometry on an Accuri C6 (BD).

### Platelet activation assay

**Figure 1:**
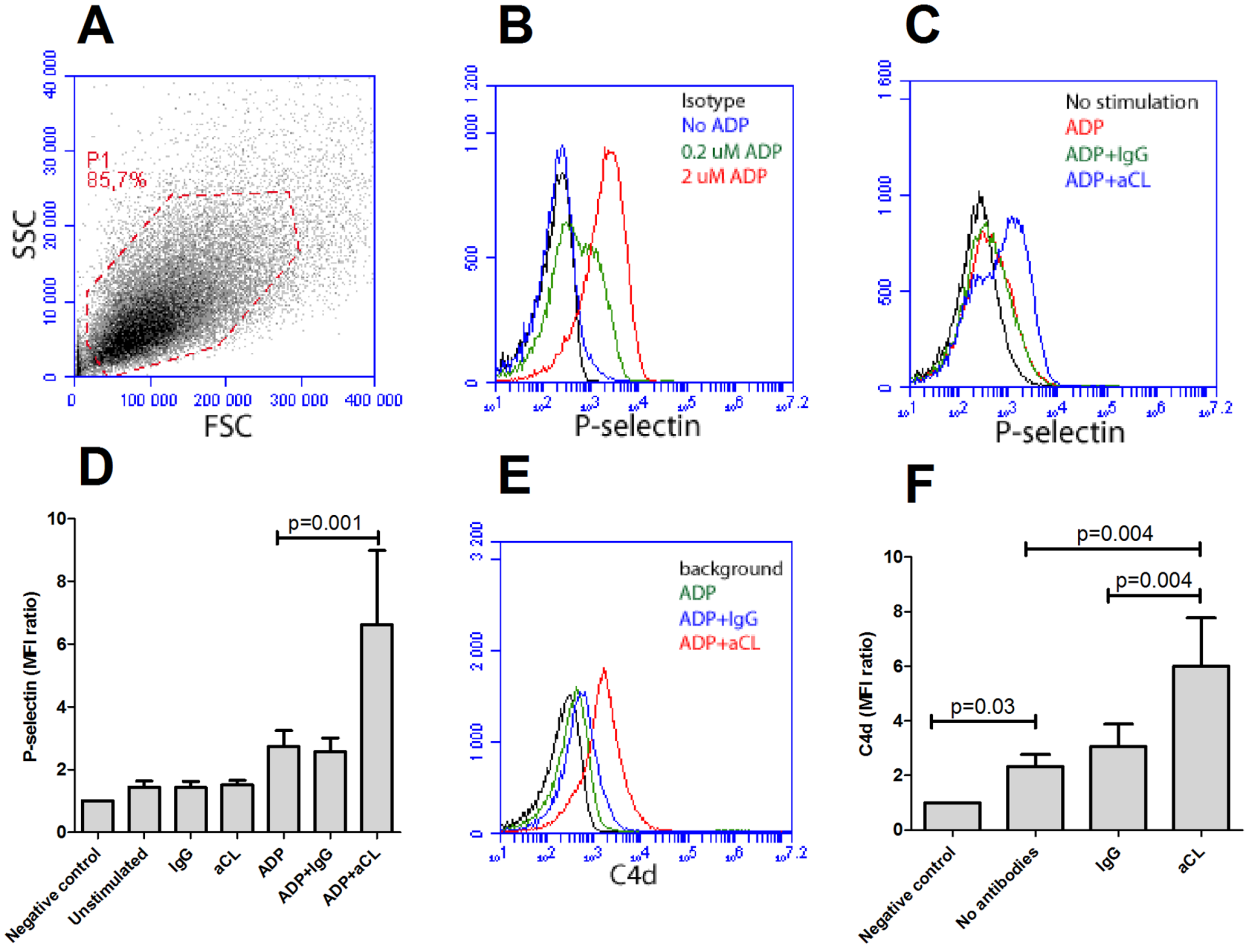
Anti-phospholipid (aPL) antibody-mediated platelet activation and complement deposition. A) Isolated platelets from healthy individuals were gated by flow cytometry (P1) and had consistently purity above 98%. B) Platelet activation after ADP stimulation was detected as P-selectin expression. The lower concentration (0.2 µM) was used for sub-optimal activation. C) Representative figure of P-selectin expression in sub-optimal activated platelets with or without addition of anti-cardiolipin antibodies (aCL). D) A summary of the data presented in Figure 1C. The results are the mean and standard deviation of six or more independent experiments. E–F) Activated and fixed platelets were incubated with or without aCL antibodies in presence of normal human serum. Complement deposition was analyzed with flow cytometry and illustrated as E) a representative histogram and F) a summary of the mean and standard deviation of six or more independent experiments.

PRP, 5 µl, was incubated with a suboptimal concentration of ADP (0.2 µM, established by titration curve (Figure 1B and Figure S1)), anti-cardiolipin IgG antibodies at a final concentration of 20 µg/ml, human IgG and PE-conjugated antibodies against P-selectin (BD) for 30 minutes at room temperature. The platelets were analyzed by flow cytometry on an Accuri C6 (BD). For detection of CD69, PRP was incubated with monoclonal antibodies against CD69 (Santa Cruz Biotechnology, Santa Cruz, CA, USA), in PBS for 40 min at room temperature. The platelets were washed once in PBS and then incubated with FITC-conjugated rabbit anti-mouse Ig antibodies (Dako) for an additional 30 min at 4°C. The platelets were analyzed by flow cytometry on an Accuri C6 (BD).

### Statistics

Spearman's correlation test was used to analyze correlations between C1q and C4d deposition on platelets. For paired analyses, Friedman test was followed by Wilcoxon matched-pairs signed rank test. For group analyses, Kruskal-Wallis test was followed by Mann-Whitney U test. Bonferroni correction was used as a post hoc test for all analyses. Platelet deposition of C1q and C4d did not assume Gaussian distribution why the variables were normalized through logarithms and associations determined by logarithmic regression analysis. The odds ratio (OR) describes the OR for the investigated variable if it increases by one standard deviation. The cut-off for high C1q and C4d deposition on platelets was determined by the 95 percentile of the healthy individuals. A p-value <0.05 was considered statistically significant.

## Results

### Anti-cardiolipin antibodies mediate platelet activation and complement deposition in vitro

Studies have shown that aPL antibodies can interact with platelets and amplify platelet activation [10], [11], [12], [13], [14], [15]. However, it is not known whether or not aPL antibodies contribute to complement activation on platelets. In this study, isolated platelets were first incubated with anti-cardiolipin (aCL) antibodies, or human IgG, and P-selectin expression measured by flow cytometry as a marker of platelet activation. Using sub-optimally ADP-activated platelets, this study found that aCL antibodies, but not purified human IgG, were able to amplify platelet activation (p = 0.001, Figures 1C and 1D). However, this effect was not seen in non-activated platelets, indicating that low grade platelet activation was necessary to allow aCL antibody interactions with the platelets (Figure 1D). Thus, the data presented herein validated the methodology used and supports the observation that aCL antibodies were able to amplify platelet activation, which is in agreement with previous investigations [10], [11], [12], [13], [14], [15].

In addition, the ability of aCL antibodies to support complement activation on platelets was tested. Purified platelets were activated with ADP and subsequently fixed with paraformaldehyde to end the activation process. The fixation of the platelets also prevented extensive complement-mediated lysis of the activated platelets during the course of the experiment. Once activated and fixed, serum from a healthy individual supplemented with either human IgG or aCL antibodies was added. Addition of aCL antibodies, but not human IgG, markedly increased the C4d deposition on activated fixed platelets (p = 0.004, Figures 1E and 1F). Even in the absence of additional antibodies, using human serum from a healthy individual, the classical pathway of the complement system was activated and C4d was readily measured on the surface of activated platelets (p = 0.03, Figure 1F). Thus, in vitro, activated platelets supported classical pathway activation and subsequent deposition of C4d and this process was amplified in the presence of aCL antibodies.

### Anti-phospholipid antibody-mediated complement deposition in SLE patients

**Figure 2:**
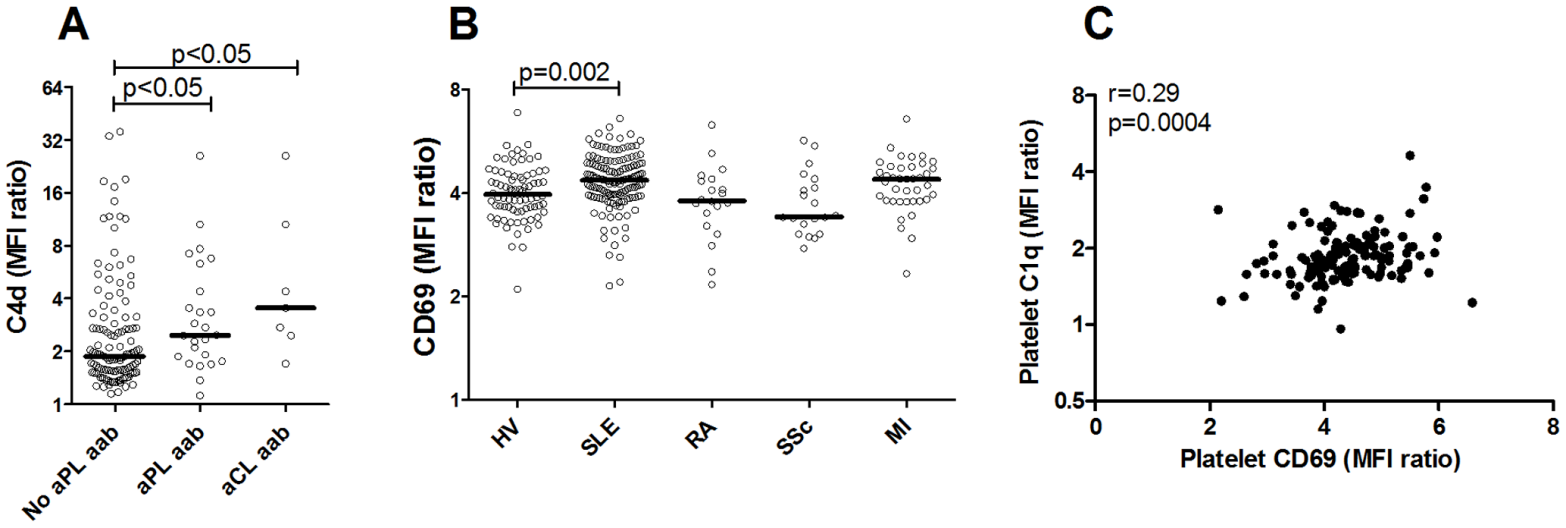
Complement deposition on platelets in SLE patients is associated with aCL antibodies and platelet activation. A) Platelet expression of C4d was analyzed in 148 SLE patients and grouped into patients with or without anti-phospholipid (aPL) or anti-cardiolipin (aCL) antibodies at the time of blood sampling. The line represents the median value in each group. B) Platelet expression of CD69 was measured by flow cytometry in healthy volunteers (HV), patients with systemic lupus erythematosus (SLE), rheumatoid arthritis (RA), systemic sclerosis (SSc) and myocardial infarction (MI). The line represents the median value in each group. C) Correlation analysis between C1q deposition on platelets and platelet activation marker CD69 in the 148 SLE patients.

The presence of aPL antibodies has previously been statistically associated with complement deposition on platelets in SLE patients [22], [23], [24], but to date it has not been established whether aPL antibodies truly support complement activation on platelets in SLE patients. At the time-point of blood sampling, 25/148 SLE patients (17%) had anti-cardiolipin or anti-B2GP1 antibodies of IgG type, or presence of lupus anticoagulant (LA). Presence of any of these factors was associated with increased C4d deposition on platelets (p<0.05, Figure 2A). When analyzed separately, anti-cardiolipin antibodies were associated with increased C4d deposition on platelets (p<0.05 Figure 2A). Using a cut-off for high levels of C4d deposition on platelets, presence of anti-cardiolipin antibodies at the time point of blood sampling was highly associated with increased C4d deposition on platelets (OR = 7.1 (1.1–44.3), p = 0.04).

However, patients without any detectable levels of aPL antibodies also had high levels of C4d on platelets. Thus, aPL antibodies did not explain all complement deposition on platelets, but clearly other factors also contributed. As described in Figure 1F, as well as previously [23], [26], platelet activation, even in the absence of autoantibodies, supported complement activation. SLE patients are known to have increased platelet activation and accordingly, increased platelet expression of CD69 (p = 0.002, Figure 2B) was demonstrated in this study, which correlated to complement deposition on platelets (r = 0.29, p = 0.0004, Figure 2C). Thus, our results suggest that both platelet activation and aPL antibodies can act synergistically to support complement deposition and activation on platelets in SLE patients.

### Complement deposition on platelets is associated with venous, but not arterial, thrombosis in SLE patients

In SLE patients, increased C4d deposition on platelets has been suggested to be associated with vascular events [23], [24], [28]. However, there are discrepancies in these investigations as to which vascular events, venous or arterial, complement deposition on platelets is associated.

In this study we observed no associations between C1q and C4d deposition on platelets and arterial vascular disease including myocardial infarction (OR = 1.2 (0.4–3.9), p = 0.81 and OR = 2.4 (0.7–8.1), p = 0.16, respectively) and cerebrovascular insult (OR = 0.8 (0.4–1.5), p = 0.44 and OR = 0.4 (0.1–1.2), p = 0.09, respectively, Table 4) in SLE patients using a logistic regression model adjusting for traditional cardiovascular risk factors. Not even when combining all arterial vascular diseases (claudicatio intermittens, angina pectoris, myocardial infarction and cerebrovascular insult) could an association with C1q or C4d deposition on platelets be found (OR = 0.8 (0.5–1.4), p = 0.50 and OR = 0.7 (0.4–1.3), p = 0.23, respectively, Table 4). Thus, in this study's SLE cohort, complement deposition on platelets was not associated with arterial vascular events. However, both C1q and C4d deposition on platelets were clearly associated with deep venous thrombosis (OR = 2.3 (1.2–4.2), p = 0.008, and OR = 2.0 (1.2–3.4), p = 0.01, respectively), independent of traditional cardiovascular risk factors: age, gender, smoking, hypertension, hyperglycemia, diabetes and obesity. Combining all venous manifestations the association with C1q and C4d levels on platelets remained statistically significant (p = 0.04 and p = 0.03, respectively, Table 4). For both arterial and venous thrombosis, adjusting for presence of aPL antibodies in the logistic regression model did not affect the results significantly (Table 4).

**Table 4:** Associations between complement deposition on platelets and cardiovascular disease and venous thromboembolism.

Manifestation	N		OR (95% CI)	p-value	OR (95% CI)^a^	p-value^a^	OR (95%CI)^d^	p-value^d^
Arterial^b^	25	C1q	1.0 (0.7–1.5)	0.98	0.8 (0.5–1.4)	0.50	0.8 (0.5–1.4)	0.40
		C4d	0.8 (0.5–1.3)	0.28	0.7 (0.4–1.3)	0.23	0.6 (0.3–1.2)	0.18
CVI	14	C1q	0.9 (0.5–1.6)	0.75	0.8 (0.4–1.5)	0.44	0.7 (0.4–1.3)	0.23
		C4d	0.5 (0.2–1.2)	0.12	0.4 (0.1–1.2)	0.09	0.3 (0.1–1.0)	**<0.05**
MI	8	C1q	1.0 (0.5–2.1)	0.92	1.2 (0.4–3.9)	0.81	2.0 (0.4–11.6)	0.42
		C4d	1.3 (0.7–2.4)	0.44	2.4 (0.7–8.1)	0.16	2.9 (0.8–11.5)	0.12
DVT	12	C1q	2.2 (1.3–3.8)	**0.005**	2.3 (1.2–4.2)	**0.008**	2.2 (1.2–4.2)	**0.01**
		C4d	1.7 (1.0–2.7)	**0.04**	2.0 (1.2–3.4)	**0.01**	2.0 (1.1–3.3)	**0.01**
Venous c	17	C1q	1.7 (1.0–2.7)	**0.03**	1.7 (1.0–2.9)	**0.04**	1.7 (1.0–2.9)	**0.04**
		C4d	1.4 (0.9–2.2)	0.11	1.7 (1.1–2.8)	**0.03**	1.7 (1.1–2.8)	**0.03**

a Adjusted for traditional risk factors; age, gender, smoking, hypertension, hyperglycemia and diabetes.

b Arterial disease includes cerebrovascular insult (CVI), myocardial infarction (MI), angina pectoris and claudicatio intermittens.

c Venous thrombosis includes deep venous thrombosis (DVT) and pulmonary embolism.

d Further adjusted for presence of aPL antibodies at time-point of blood sampling.

Whereas our statistical model uses a continuous variable, other investigators have used a cut-off to describe complement deposition on platelets as a dichotomized variable [22], [28]. Using the cut-off as described in the material and methods section C4d deposition on platelets still remained highly associated with venous thrombosis (OR = 5.3 (1.3–22.1), p = 0.02) after adjusting for traditional risk factors and aPL antibodies. However, using the same cut-off, C4d deposition on platelets was associated with a decreased frequency of arterial thrombosis (OR = 0.2 (0.1–0.8), p = 0.03) as well as stroke (OR = 0.2 (0.0–1.0), p<0.05), adjusted for traditional cardiovascular risk factors and presence of aPL antibodies. Thus, in SLE patients, increased complement deposition on platelets was clearly associated with venous, and not arterial, thrombosis independently of traditional cardiovascular risk factors and aPL antibodies.

### Complement deposition on platelets is not specific for SLE patients

**Figure 3:**
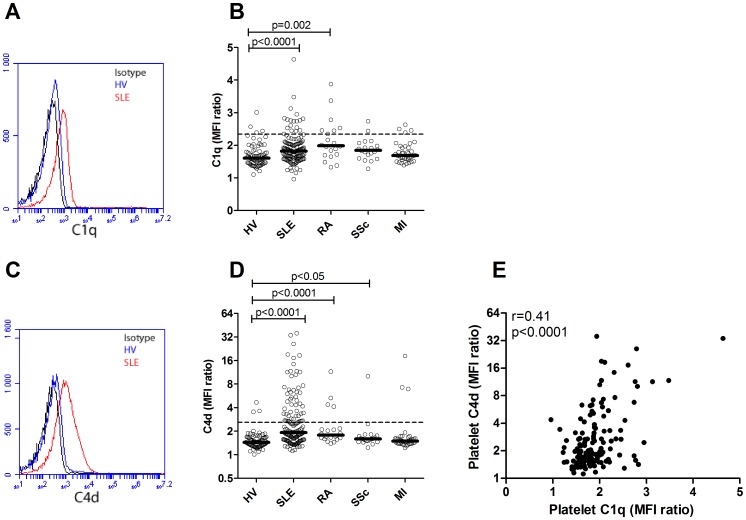
Increased complement deposition on platelets in SLE patients. A) Deposition of C1q and C) C4d was analyzed by flow cytometry and illustrated as representative flow cytometry histograms. Deposition of B) C1q and D) C4d on platelets from healthy volunteers (HV), patients with systemic lupus erythematosus (SLE), rheumatoid arthritis (RA), systemic sclerosis (SSc) and myocardial infarction (MI). The dotted lines represent the 95^th^ percentile of the healthy volunteers and depict the cut-off level for positivity in each analysis. E) Correlation analysis between C1q and C4d deposition on platelets in the SLE patients.

C4d deposition on platelets has been suggested to be highly specific for SLE [22]. However, whether C1q deposition on platelets is specific for SLE had not been investigated previously. Complement deposition of both C1q and C4 on platelets were markedly increased in SLE patients as compared to healthy volunteers (p<0.0001 for both analyses, Figure 3). Patients with rheumatoid arthritis had increased C1q deposition (p = 0.002, Figure 3B) as well as increased C4d deposition (p<0.0001, Figure 3D) whereas patients with systemic sclerosis only were found to have increased C4d deposition (p<0.05, Figure 3D) on platelets as compared to healthy volunteers. Notably, some of the apparently healthy individuals had increased C4d deposition on their platelets. Using the cut-off value for high and low complement deposition on platelets 12% of the SLE patients, 35% of the rheumatoid arthritis patients, 10% of the systemic sclerosis patients, 12% of the myocardial infarction patients and 5% of the healthy individuals were regarded as having high levels of C1q on platelets (Figure 3B). For the C4d deposition on platelets, 35% of the SLE patients, 20% of the rheumatoid arthritis patients, 5% of the systemic sclerosis patients, 8% of the myocardial infarction patients and 4% of the healthy individuals were regarded as having high levels (Figure 3D). There was a correlation between C1q and C4d deposition on platelets (r = 0.41, p<0.0001, Figure 3E). Only 67% of the SLE patients positive for C1q deposition were also positive for C4d deposition on platelets suggesting that complement activation does not always proceed after C1q binding. Furthermore, of the SLE patients negative for C1q deposition, 31% had increased deposition of C4d on platelets, indicating that small amounts of C1q might be enough to activate C4.

### Complement deposition on platelets is associated with disease activity

**Figure 4:**
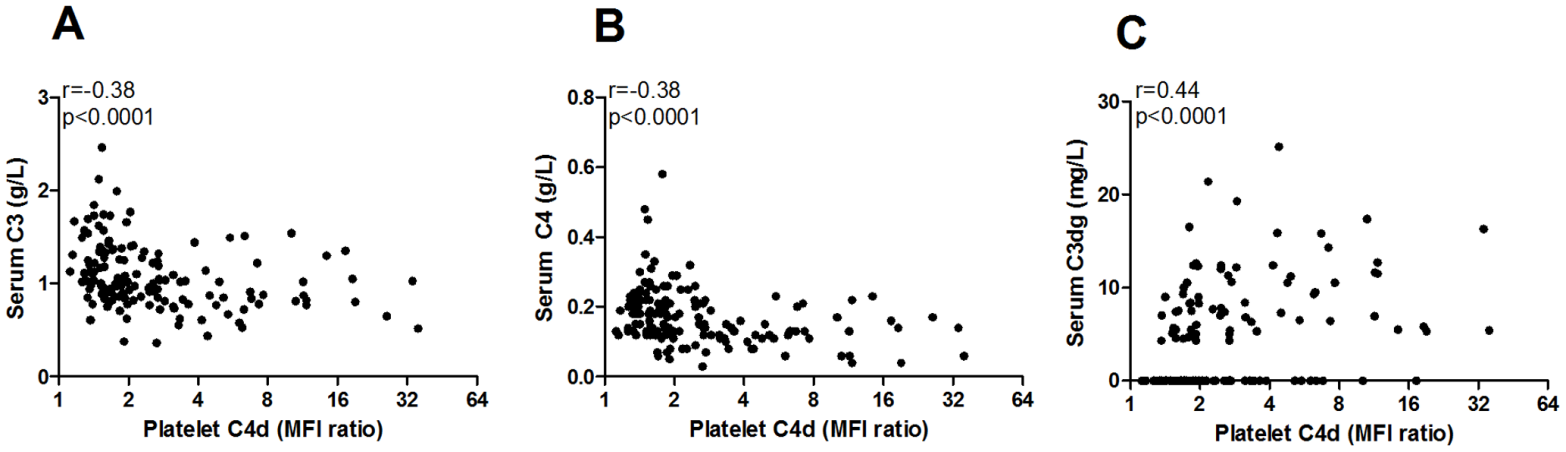
Platelet C4d deposition is associated with complement consumption and activation. C4d deposition on platelets from SLE patients was correlated to A) serum level of C3, B) serum level of C4, and C) the complement activation split fragment C3dg.

To investigate the clinical relevance of our findings we first assessed if complement deposition on platelets was associated with disease activity. C4d deposition on platelets, but not C1q deposition, was positively correlated to SLEDAI (r = 0.27, p = 0.001). Furthermore, patients with active disease (SLEDAI≥5) had highly increased C4d deposition on their platelets (p = 0.008) compared to SLE patients with no or low disease activity. Patients treated with Prednisone had a higher C4d deposition on platelets (p = 0.006), most likely due to increased disease activity seen in this group (p = 0.01). None of the other immunosuppressive treatments affected C1q or C4d deposition on platelets in a statistically significant manner. Even though not correlated to disease activity in general, C1q deposition on platelets was increased in SLE patients with ongoing arthritis (p = 0.01). For C4d deposition, no associations were found with any specific clinical disease manifestation. Instead C4d deposition correlated with the presence in serum of anti-dsDNA antibodies (p = 0.03) and low levels of either C3 or C4 (p<0.0001). The deposition of C4d on platelets was inversely correlated with serum levels of both C3 and C4 (r = −0.38, p<0.0001 for both analyses, Figures 4A and 4B) as well as positively correlated with the complement split product C3dg (r = 0.44, p<0.0001, Figure 4C). Finally, even when using a modified SLEDAI excluding any score for anti-dsDNA antibodies or low complement levels, C4d deposition on platelets remained statistically significantly correlated to disease activity, even though the association was weak (r = 0.17, p = 0.04).

## Discussion

Anti-phospholipid antibodies are well-known important pro-thrombotic factors contributing to development of venous thrombosis and stroke in SLE patients [9], [34], [35]. The molecular mechanism of how aPL antibodies mediate development of thrombosis is not fully understood but may involve activation of both platelets and the classical pathway of the complement system [16], [17], [23]. In human C2 deficiency (C2D), anti-cardiolipin antibodies are frequently seen but virtually never lead to development of venous thrombosis [17]. Furthermore, in mouse, C3, C5a and C6 are all necessary for development of aPL antibody-mediated thrombosis [16], [18], [19], [20], [21]. In this investigation we have studied the role of aPL antibodies in mediating complement activation on the surface of platelets and if this could be a possible mechanism linking aPL antibodies, complement activation, platelet activation and vascular events in SLE patients. Furthermore, we present a detailed examination of associations between complement deposition on platelets and other clinical variables.

Increased complement activation has been seen on platelets in SLE patients, especially in patients with aPL antibodies [22], [23], [24]. However, it was not known if aPL antibodies could support complement activation on platelets. Data presented herein demonstrates that aPL antibodies indeed allow complement activation on platelets by two separate mechanisms, both of which could be operating in SLE patients. Firstly, aPL antibodies contribute to platelet activation-mediated complement deposition. It is well-established that aPL antibodies amplify platelet activation [10], [11], [12], [13], [14], [15], which was verified in this investigation. Activated platelets expose several molecules including phosphatidylserine and chondroitinsulfate which support binding of C1q and subsequent complement activation [26]. Supporting the hypothesis of platelet activation being sufficient to allow complement activation we observed that sera from healthy individuals supported complement activation on the surface of activated platelets (Figure 1F) also confirming observations in one of our previous studies [23].

Secondly, we hypothesized that the complement-fixing ability of some anti-PL antibodies [25] may allow C1q binding with subsequent activation of the classical pathway on platelets. To test the validity of this model, normal human serum, supplemented with purified aPL antibodies, was added to activated fixed platelets. Using this experimental approach it was found that aPL antibodies also mediated complement activation on platelets independently of their ability to also support platelet activation. Those results are strongly supported by the current as well as previous investigations demonstrating associations between aPL antibodies and complement deposition on platelets [22], [23], [24]. Thus, we suggest that aPL antibodies, through both platelet activation and binding of complement-fixing antibodies, support complement activation on platelets.

However, aPL antibodies are not indispensable in activating the complement system on platelets, and several mechanisms may operate to mediate complement activation on platelets. This was highlighted by the significant number of SLE patients having no detectable aPL antibodies but still having high levels of both C1q and C4d on platelets. One explanation for this may be presence of other anti-platelet antibodies, including anti-GPIIb/IIIa [36], [37], [38], but more likely, complement deposition on platelets can be explained by increased platelet activation. In this study we could demonstrate that SLE patients had increased platelet activation and the platelet activation correlated with complement deposition on the platelet surface. The cause for the initial platelet activation in SLE is not known but may include immune complexes, shear stress, type I IFNs or endothelial damage with exposure of extracellular matrix proteins and collagen [39], [40], [41]. Furthermore, oxidized LDL (oxLDL), which is increased in SLE patients [42], may also participate in the initial platelet activation [43]. Thus, based on our results, we suggest that complement deposition is increased in SLE patients due to ongoing platelet activation and this process, both platelet activation and complement activation on platelets, is amplified in the presence of aPL antibodies.

Earlier studies have established that anti-PL antibodies are associated with development of venous thrombosis and stroke in SLE patients [9], and previous studies have demonstrated an association between increased complement deposition on platelets and vascular events [23], [24], [28]. However, there are some discrepancies in the literature with regard to which type of vascular event, venous or arterial, complement deposition on platelets is associated with. Furthermore, none of the previous studies have taken into account the role of traditional cardiovascular risk factors in their statistical analyses. In the current investigation we found that complement deposition on platelets was associated with venous, but not arterial, thrombosis, which is in line with our previous study [23]. However, in this study, data demonstrated that the association to venous thrombosis was independent of traditional cardiovascular risk factors and aPL antibodies. Previous studies have suggested that aPL antibodies found in patients with venous thrombosis have increased complement-fixing ability compared to aPL antibodies found in patients with arterial thrombosis [25] and this may be one reason for the increased complement deposition on platelets in patients with aPL antibodies and venous thrombosis.

C4d deposition on platelets has been suggested to be highly specific for SLE [22] but it was not known if C1q deposition on platelets could be seen in inflammatory diseases other than SLE. In contrast to a previous investigation [22] increased C4d and C1q deposition could be readily observed on platelets in patients with rheumatoid arthritis, increased C4d deposition on platelets was found in patients with systemic sclerosis, as well as high levels of complement deposition found on platelets in some apparently healthy individuals. Thus, complement activation on platelets is not specific for SLE but associated with platelet activation in general. However, different patterns of C1q and C4d deposition were found in SLE patients and patients with rheumatoid arthritis. Patients with rheumatoid arthritis had a high frequency of elevated C1q levels on platelets but a relatively low frequency of C4d, whereas SLE patients had the opposite with high frequency of elevated C4d levels compared to a relatively low frequency of C1q. This suggests that different mechanisms of complement activation and regulation might be operating in the two diseases. Interestingly, SLE patients with ongoing arthritis had increased C1q deposition on platelets compared to SLE patients with no arthritis. Even though the pathogenesis of arthritis is different between rheumatoid arthritis and lupus, platelet activation has been demonstrated in the joints of patients with rheumatoid arthritis, but the contribution of complement activation on platelets to this is not known [44]. Further studies are needed to elucidate how complement activation on platelets is regulated in different conditions and contributes to disease manifestations.

In conclusion, we suggest that aPL antibodies are able to amplify C4d deposition on platelets through two separate mechanisms; amplification of platelet activation, and providing complement-fixing antibodies on platelets. Complement deposition on platelets is associated with venous, but not arterial, thrombosis in SLE patients, independent of traditional cardiovascular risk factors and aPL antibodies. Further studies are needed to elucidate the underlying mechanisms linking complement activation on platelets to cardiovascular disease.

## Supporting Information

Figure S1 **Dose-response curve for ADP.** ADP, at different concentrations, was incubated with platelets for 15 minutes at room temperature and platelet activation analyzed by P-selectin expression by flow cytometry. Concentrations ranging between 0.2-0.4 µM ADP were found to induce sub-optimal platelet activation.
(TIF)
