# Supplementary figures and images for: Platelet Activation and Anti-Phospholipid Antibodies Collaborate in the Activation of the Complement System on Platelets in Systemic Lupus Erythematosus
Source: PLoS One. 2014 Jun 12;9(6):e99386. doi: 10.1371/journal.pone.0099386 (PMC4055750; doi:10.1371/journal.pone.0099386)

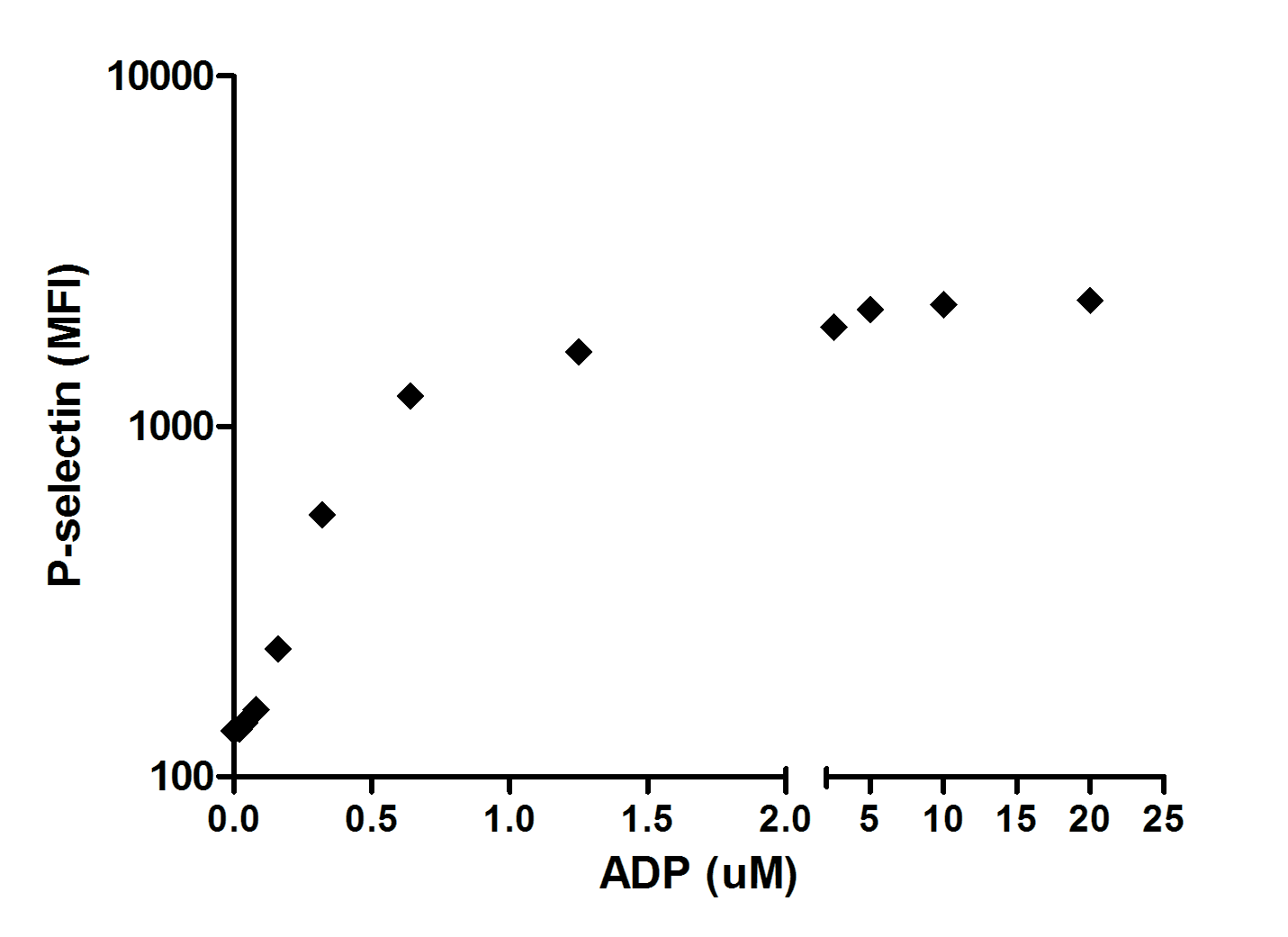

Supplement: Figure S1 — Dose-response curve for ADP. ADP, at different concentrations, was incubated with platelets for 15 minutes at room temperature and platelet activation analyzed by P-selectin expression by flow cytometry. Concentrations ranging between 0.2-0.4 µM ADP were found to induce sub-optimal platelet activation. (TIF) [file pone.0099386.s001.tif]
